# Reviewed article: Azevedo LS, Oliveira NN, Oliveira RR. Reports, excess risk and spatial distribution of domestic violence against women, Brazil, 2015-2020. Epidemiol Serv Saude. 2025:34;e20240277

**DOI:** 10.1590/S2237-96222025v34e20240277.a

**Published:** 2025-07-11

**Authors:** Jaqueline Arboit

**Affiliations:** UFSM, departamento de Ciências da Saúde

**Table:** 

Rating	Excellent	Good	Average	Below average	Poor
Interest		✓			
Quality		✓			
Originality		✓			
Overall		✓			

**Table:** 

Questionnaire	Yes	No	Not applicable
Does the manuscript contain new and significant information to justify publication?	✓		
Does the Abstract (Summary) clearly and accurately describe the content of the article?	✓	✓	
Is the problem significant and concisely stated?			
Are the methods described comprehensively?	✓		
Are the interpretations and conclusions justified by the results?	✓		
Is adequate reference made to other work in the field?		✓	

## Manuscript Structure

Length of article is: Adequate

Number of tables is: Adequate

Number of figures is: Adequate

Please state any conflict(s) of interest that you have in relation to the review of this paper (state “none” if this is not applicable): None

## Comments

Prezados (as) autores (as),

Primeiramente gostaria de parabenizá-los pela escolha da temática que é relevante e reflete uma problemática presente no cotidiano de tantas mulheres em nosso país. No decorrer da leitura, foram encontradas algumas fragilidades, que necessitam ser revisadas a fim de contribuírem para possível publicação. A seguir serão descritas as sugestões de alterações.

Título:

1. Não contempla adequadamente o conteúdo do manuscrito. Sugere-se: Notificações, excesso de risco e distribuição espacial da violência doméstica contra as mulheres, Brasil, 2015 a 2020.

Justifica-se a inserção de “notificações e excesso de risco”, visto que apenas a expressão “distribuição espacial” não dá a dimensão da análise desenvolvida pelos autores.

Justifica-se a substituição de mulher (singular) por mulheres (plural), pois a palavra no plural é empregada objetivando proporcionar visibilidade às heterogeneidades de natureza étnica, geracional, de orientação sexual, de deficiência e de inserção social, econômica e regional presentes entre as mulheres, em convergência com as considerações da Política Nacional de Enfrentamento à Violência contra as Mulheres. Assim, indica-se usar mulher no plural, ao longo de todo o manuscrito.

Resumo:

1. Objetivo – reescrever: analisar as notificações, o excesso de risco e a distribuição espacial da violência doméstica contra as mulheres no Brasil, no período de 2015 a 2020.

2. Quanto ao método: deixar explícito que se trata de um estudo transversal, descritivo.

Descritores:

1. Suprimir “Violência de gênero” e adicionar “Sistemas de Informação em Saúde”.

Introdução:

1. Não apresenta uma sequência lógica de ideias. Atentar para apresentar dados epidemiológicos a nível global e após, nacional. O primeiro parágrafo, por exemplo, traz dados epidemiológicos relativos ao Brasil. O segundo contextualiza a violência contra a mulher, apresenta um conceito de violência doméstica, e novamente traz dados epidemiológicos, a nível mundial. A redação dessa forma prejudica a leitura fluída e compreensão pelo leitor.

2. Esta seção deve apresentar o estado da arte e justificar o desenvolvimento da pesquisa. Contudo, os autores empregaram 11 referências, das quais apenas três são oriundas de artigos em periódicos (nenhum deles de impacto internacional). Considera-se fundamental ampliar qualitativamente as referências dessa seção com a inclusão de referências internacionais oriundas de periódicos. Além disso, estamos em 2024 e a produção na temática da violência contra as mulheres é passível de ser recuperada, não sendo adequado manter referências de artigos de 2017 e 2018, por exemplo.

3. Do mesmo modo, outras referências da introdução são passíveis de atualização. Um exemplo é a referência 1 - Anuário Brasileiro de Segurança Pública de 2020. Atualmente, está disponível o Anuário Brasileiro de Segurança Pública de 2023, com diversos dados relacionados as mulheres. Link: https://forumseguranca.org.br/wp-content/uploads/2023/07/anuario-2023.pdf

3. Rever a afirmação “observa-se a escassez de estudos sobre o agravo em nível de Brasil, especialmente, abordando suas taxas, excesso de risco e distribuição espacial...”. Os autores realizaram um levantamento de estudos em bases de dados nacionais e internacionais para identificar essa escassez?

3. Quanto ao objetivo, reescrever: analisar as notificações, o excesso de risco e a distribuição espacial da violência doméstica contra as mulheres no Brasil, no período de 2015 a 2020.

Método:

1. Estruturar a seção em tópicos para facilitar a compreensão do leitor. Inclusive, esta organização está prevista nas normas do periódico.

2. Deixar claro que se trata de estudo transversal, descritivo.

3. Em relação aos critérios de inclusão, analisar a possibilidade de incluir vítimas do sexo feminino de 18 a 59 anos, ou seja, apenas mulheres adultas; excluindo-se assim, crianças, adolescentes e idosas. Essa sugestão se deve ao fato de que as adolescentes (12 a 18 anos segundo o ECA) também estão contempladas pelas diretrizes desse Estatuto. Além disso, as situações de violência que as adolescentes vivenciam se assemelham, muitas vezes, mais as formas de violência vividas pelas crianças do que pelas mulheres adultas, o que pode ser identificado na literatura. Portanto, é necessário que os estudos considerem especificamente esse recorte de faixa etária (crianças e adolescentes, mulheres adultas, idosas). Caso contrário, há risco de viés na interpretação dos resultados.

4. Consta no site do periódico em “Orientações para preparação do texto”, que anglicismos, devem ser evitados, optando por termo no vernáculo (ex.: “dados faltantes” ao invés de “missing”). Observar essa orientação e rever a redação na linha 20-21 desta seção.

Resultados:

1. Rever essa seção considerando a sugestão do método de “incluir apenas vítimas do sexo feminino de 18 a 59 anos”.

2. Se possível, acrescentar uma tabela/figura com os dados relativos ao excesso de risco de violência nos estados.

Discussão:

1. Os autores precisam deixar claro nesta seção quais os novos achados e como este estudo contribui para o avanço do conhecimento na área, visto que existem outros estudos publicados que abordam as notificações da violência contra as mulheres no Brasil.

2. Embora esteja bem organizada, apresente semelhanças e contrapontos entre estudos; os autores tem dificuldade de explorar/aprofundar alguns aspectos da discussão. Por exemplo: Em relação a esta frase - “Assim, os mapas ajudam profissionais a identificarem problemas em suas regiões de atuação, contribuindo para a tomada de decisões.” Como os mapas podem auxiliar nessa tomada de decisões?

Outro exemplo: “Este estudo é uma importante fonte de dados e trouxe resultados relevantes sobre o cenário do agravo...”. Quais os novos achados que justificam a publicação?

3. Assim como na introdução, a literatura internacional é pouco explorada nessa seção.

Referências

1. Os autores utilizam apenas 30 referências, sendo que a revista permite a utilização de até 40 referências para esta categoria de manuscrito. Nesta perspectiva, os autores podem buscar mais estudos nacionais e internacionais atualizados para agregar a introdução e discussão.

***O texto necessita de revisão de língua portuguesa (sintaxe, concordância e pontuação).

